# Enhancing Self-Reported Assessment of Working Conditions in Policing: Revisiting the Psychometric Properties and Applications of the Police Stress Questionnaire

**DOI:** 10.1177/00332941231207957

**Published:** 2023-10-30

**Authors:** Lillis Rabbing, Bjørn Lau, Knut Inge Fostervold, John Blenkinsopp, Brita Bjørkelo

**Affiliations:** Doctoral Research Fellow, 6274 Norwegian Police University College, Norway; Department of Psychology, University of Oslo, Norway; Department of Psychology, 6305 University of Oslo, Norway; Department of Psychology, 6305 University of Oslo, Oslo, Norway; Department of Psychology, 289185 Oslo New University College, Oslo, Norway; 6274 Norwegian Police University College, Oslo, Norway; 289185 Oslo New University College, Oslo, Norway

**Keywords:** Police spesific measures, police stress questionnaire, PSQ, sworn police officers, employees without police education, civilian, psychometric properties, not applicable, work stess, working conditions

## Abstract

**Objectives:**

Policing is recognized as a highly stressful occupation, encompassing stressors not commonly encountered in other fields. In response, police-specific stress scales have been developed and used when studying police work. Despite changes in the composition of police personnel, most studies examining police working conditions focus on sworn police officers (SPO), excluding employees without police education (EWPE). To advance research and practice on stress in the police, align results, and increase the possibilities for comparisons across studies using police-specific measures (PSMs) we conducted a psychometric evaluation of the two scales in the Police Stress Questionnaire (PSQ). We examined whether adding “Not Applicable” to the response scales would reduce vulnerability and make the PSQ more robust.

**Method:**

Based on a survey with a randomised sample (*N* = 560) of SPO and EWPE in the Norwegian Police, we tested the original factor structures of the PSQ through Confirmatory Factor Analysis including tests of factor structures from previous studies.

**Results:**

For all models, the indicators of fit indicated a poor fit with either our whole or stratified sample. The response choice ‘Not Applicable’ provided extended information for SPOs and EWPEs on the PSQ.

**Conclusions:**

To promote aligning results and enabling comparisons across studies using the PSQ, we suggest treating the PSQ scales as formative indexes, rather than reflective scales. Adding “Not Applicable” to the response scale offers an influential elaboration of the PSQ with beneficial and extended information. Generalised studies of stress in the police should include the entire population working there.

## Background

Work-related stress has long been recognised as a public health issue (Siegrist, 2002) with a particular focus on occupations identified as especially stressful. A significant body of research recognises policing as a highly stressful occupation with consequences for employees’ mental and physical health, performance, and interactions with citizens (McCarty et al., 2019; Queriós et al., 2020), leading researchers to describe police mental health as a public health concern. While there are substantial structural and cultural differences both internally within countries and externally across different countries and areas, there is a global trend toward civilianisation in the police sector (Adams & Mastracci, 2020; Conor et al., 2019; Forst, 2000; Kiedrowski, 2019; Lentz et al., 2020; McCarty & Skogan, 2013; Varker et al., 2022). The composition of police employees has consequently shifted from predominantly sworn police officers (SPO) to include a large proportion of employees without police education (EWPE) (Conor, 2019; Ellison, 2004; Lentz et al., 2020). The earlier stages of civilianisation involved employing EWPE to perform non-policing work (e.g., IT support, finance, HR) (Orosco & Gaub, 2022). EWPE today are hired with professional skills as e.g., lawyers, cyber technicians, psychologists, computer engineers, criminologists, or biologists, undertaking work which is unmistakeably police work in units like forensics, cybercrime, intelligence, analysis, or crime investigations (Jackman et al., 2021; Varker et al., 2022). EWPE in these positions encounter the same stressors as SPO (Lentz et al., 2020) yet research on stress in the police that includes both groups is rare (Adams & Mastracci, 2020; Lentz et al., 2020; Orosco & Gaub, 2022; Varker et al., 2022).

To provide greater credibility and a more comprehensive and updated understanding of working conditions in the police we need studies encompassing all police employees (Jackman et al., 2021; Orosco & Gaub, 2022). Since studies of police stress often apply context-sensitive police-specific measures (PSM) developed by scholars, for and by SPO, to gauge the most salient operational and/or organisational stressors of policing (Rabbing et al., 2022; Shane, 2010) most research on stress in the police has applied PSM without including EWPE. Consequently, little is known about how work-related factors impact EWPE. Providing better measures and suitable samples is therefore a contribution to advancing research on stress in policing.

It is crucial to understand how the PSM work when expanded to include EWPE. In this study, we investigated the psychometric properties of the most widely used PSM globally (Delgado Ramos & Vélez Vega, 2022; Jackman et al., 2021; McCreary & Thompson, 2006), the Police Stress Questionnaire (PSQ) (McCreary & Thompson, 2006). Despite being designed exclusively for SPO the PSQ has been applied to both SPO and EWPE in a few studies (Jackman et al., 2021; Short, 2021; Varker et al., 2022).

The PSQ consists of two independent 20-item scales; the Operational Police Stress Questionnaire (PSQ-Op) measuring operational stress, i.e. stressors associated with doing the job, and the Organisational Police Stress Questionnaire (PSQ-Org) measuring organisational stress, i.e. ‘stressors associated with the organisation and the culture within which they are performing their job’ (McCreary & Thompson, 2006, p. 499). Both scales measure the severity of the stressors, using a 7-point Likert response scale ranging from 1 (‘not at all stressful’) to 7 (‘very stressful’), with 4 indicating moderate stress (McCreary & Thompson, 2006).

The PSQ was developed inductively by 55 experienced SPO in Canada who identified stressful characteristics of their work, the impact of job-related stress on their families, and the effects of stress at home on their job performance and health (McCreary et al., 2017; McCreary & Thompson, 2006). The relevance of the items in each scale was subsequently assessed by having 47 SPO rate each item on severity and frequency. Cronbach’s alpha reliability coefficients were computed for both scales and the corrected item-total correlation was used to verify item contribution. The stressor ‘Shift work’ did not meet the threshold of α>.30 but was kept in the scale due to its significance in policing (McCreary & Thompson, 2006). The instrument has good construct, discriminant, and concurrent validity (McCreary & Thompson, 2006), with low shared variance between the two scales and the severity and frequency ratings positively correlated, as well as having low shared variance with other general stress measures (McCreary & Thompson, 2006). Furthermore, the authors report PSQ is positively correlated with measures of job satisfaction (McCreary & Thompson, 2006). Other studies (Delgado Ramos & Vélez Vega, 2022; Sagar et al., 2014, 2015), confirm an acceptable convergent validity, meaning congruence with similar measures, and satisfactory concurrent validity (Rasdi et al., 2014), while matching the PSQ with other general stress measures. Most studies employing the PSQ report excellent internal consistency (α>.90) (Delgado Ramos & Vélez Vega, 2022), which should be expected for scales containing over 15 items (Taber, 2018).

However the theoretical construct, with the assumed single-factor structure of the two scales, has been questioned. PSQ’s authors recommended users conduct Exploratory Factor Analysis (EFA) to identify meaningful ‘lower-order factors’ and test a single-factor ‘higher-order’ model (McCreary & Thompson, 2006, p. 514). Following this, the factor structure has been studied by applying EFA including one of the scales (Bélanger & Blanchette, 2022; Delgado Ramos & Vélez Vega, 2022; Fayyad et al., 2020; Kukić, Subošić, et al., 2021; Li et al., 2021; Queriós et al., 2020; Shane, 2010) or selected items from one or both scales (Argo et al., 2021; Baek et al., 2021; Louw & Viviers, 2010; Queirós et al., 2020; Rasdi et al., 2014; Sagar et al., 2014, 2015). We find studies conducting Confirmatory Factor Analysis (CFA) with either one (Brunetto et al., 2022; Louw & Viviers, 2010; Queirós et al., 2020; Sagar et al., 2014, 2015; Shane, 2010) or both of the scales (Baek et al., 2021; Delgado Ramos & Vélez Vega, 2022; Kukić, Subošić, et al., 2021; Li et al., 2021), Principal Component Analysis (PCA) with either one (Fayyad et al., 2020; Louw & Viviers, 2010; Queirós et al., 2020), or both scales (Kukić, Streetman, et al., 2021; Rasdi et al., 2014; Shane, 2010), and on both scales in the Rasch Rating Scale Model (Argo et al., 2021). While including different items and different numbers of items, the findings range from showing no meaningful factor structure for the two scales (Li et al., 2021) to showing one factor for each of the two scales (Delgado Ramos & Vélez Vega, 2022; Rasdi et al., 2014). Others report either PSQ-Op or PSQ-Org have a factor structure of two (Bélanger & Blanchette, 2022; Kukić, Streetman, et al., 2021; Kukić, Subošić, et al., 2021; Queirós et al., 2020; Queriós et al., 2020), four (Louw & Viviers, 2010; Sagar et al., 2014, 2015), five (Kukić, Streetman, et al., 2021), or six factors (Fayyad et al., 2020; Shane, 2010). The literature thus provides no shared understanding of the factor structure of the PSQ-Op and PSQ-Org respectively.

Lack of shared understanding of factor structure may be explained in several ways. Not all stressors in the PSQ scales (e.g., ‘Shift work’, ‘Internal investigations’, ‘Dealing with the court’) apply to all respondents in different, countries, cultures, units or police organisations whether working as SPO or EWPE (Jackman et al., 2021). The response scale of the PSQ (i.e., 1–7) gives the respondents limited opportunities to mark non-exposure to the stressors included in the questionnaire. Additionally, the instructions for the PSQ neither clearly differentiate between exposure to and perception of the stressors, making the two scales vulnerable to a high degree of missing items, or ratings based on perceptions rather than own experiences. This in turn reduces the interpretability and the comparability of the instrument. A possible remedy, suggested by Varker et al. (2022), is to include a ‘Not applicable’ (‘N/A’) option to the response scale. With the option ‘Not applicable’, one can both avoid database deficiency (missing items) and achieve improved information. Reducing the number of missing items will increase the number of valid respondents and thus increase the statistical power and thereby the robustness of the measure. Rated as 0, the ‘N/A’ will not bias results when computed.

As lack of shared understanding of factor structure hampers the possibility of interpreting and comparing results and outcomes on stress in the police between studies and countries, we (i) undertake a psychometric evaluation of the two scales of the PSQ when applied to a sample of an entire police population, and (ii) investigate if a modification of the response scale by adding ‘Not Applicable’ would reduce vulnerability and make the scales more robust. Results will be discussed considering scale construction.

## Methods

### Participants

The Norwegian Police Service (NPS) employs the total workforce in Norway and consists of 12 police districts and units, as well as special units and the National Police Directorate (NPD). The NPS is organised and governed through the government the Ministry of Justice and Public Security, and the NPD is the highest level of authority in NPS. Based on a random sampling technique, a representative sample of 4000 NPS employees were invited to participate in the survey. Access was granted by the NPD and the sampling was conducted by the analytics unit at the NPD based on the official employee register of NPS including employees in all positions, with or without police education. Invitations to complete the online questionnaire including informed consent were sent to participants via their workplace e-mail. A total of 560 employees (SPO = 347, EWPE = 213) responded (RR: 14%). The sample showed a satisfactory representative distribution according to age (average 44.9), gender (43.2% female), and educational background (SPO/EWPE). For more details on demographics, please see Table 1.

**Table 1.** Demographics, Background Variables Among Respondents and the Population.

		*n* = 560 frequency	Per cent	Per cent invited sample	Per cent total police population
Gender	Women	242	43.2	45.4	46.2
	Men	316	56.4	54.7	53.8
	Other	2	0.4		
Marital status	Single	68	12.1		
	Married/cohabitant/partnership	455	81.3		
	Separated/divorced	30	5.4		
	Widow(er)	7	1.3		
Age (years)	−29	40	7.1	12.1	12.3
	30–39	148	26.4	30.3	30.5
	40–49	170	30.4	27.5	27.2
	50–59	167	29.8	24.6	24.4
	60 -	35	6.3	5.6	5.6
Education	Primary school	1	0.2		
	High school or vocational school	76	13.6		
	College or university (bachelor)	337	60.2		
	Higher university degree (master/PhD)	146	26.1		
Police educated	Two-year police academy	72	12.9		
	Three-year police academy, bachelor	251	44.8		
	Other	237	42.3		
Region	Urban	482	86.1		
	Rural	78	13.9		
Organisational affiliation	Special unit	152	27.1	17.7	17.8
	In a district	408	72.9	82.3	82.2
Position	EWPE/Civilian position	214	38.2		
	SPO/Police officers	346	61.8	60	59.8

### Measurement

This study is part of the ‘Police Study’ which in addition to the PSQ, included validated scales about leadership, psychological and physiological work demands, health outcomes, and resources, resilience, job engagement, and mastery of work. All scales were used without modifications except for the response scale of the PSQ (i.e., 1–7), where we added an answer category ‘Not Applicable’ (‘N/A’).

### Statistical Analysis

For preliminary analyses we used SPSS version 28. ‘N/A’ scores were treated as “missing” in descriptive statistics (see Tables 3 and 4). ANOVA tests (*p < .001*) with F-values performed to compute the probability of the mean difference between SPO and EWPE. We performed CFA using AMOS version 28, to explore the factor structures of the PSQ scales. In the CFA analyses, we tested our entire sample (*n* = 560) and stratified our sample by the position of employment (SPO, *n* = 323, and EWPE, *n* = 237). The ‘N/A’ scores were given the value of zero when computed in AMOS. The indicators used to evaluate the models' fit were Chi-square/Degrees of freedom (<5, *p < .001*), the Root Mean Square of Error Approximation (RMSEA, <.08, 95% CI), the Comparative Fit Index (CFI, >.90), and the Standardised Root Mean Square Residual (SRMR, <.08) (Boateng et al., 2018).

### Ethical Approval

This study was approved by the Regional Committee for Medical and Health Research Ethics (2020/140335), the National Centre for Research Data (project number 439242), and the NPD (ref. 201900307).

## Results

The first aim of this study was to conduct a psychometric evaluation of the PSQ (McCreary & Thompson, 2006). In these tests, we exclusively used models where all items (=20 items) in one or both scales were accounted for. Initially, a CFA was conducted on the two scales of PSQ separately. The results indicated a poor fit for all fit indicators, i.e., Chi-square/Degrees of freedom, RMSEA, CFI, and SRMR. Subsequently, a CFA was conducted where all items (=40) were loaded on a single factor. Again, the results revealed a poor fit. Moreover, we replicated and computed the different factor structures reported in previous studies (Bélanger & Blanchette, 2022; Kukić, Streetman, et al., 2021; Kukić, Subošić, et al., 2021; Queirós et al., 2020; Shane, 2010), none of which included civilians (i.e., EWPE), to look for a model fit for our whole and stratified samples, i.e., SPO and EWPE respectively. None of the factor structures reported in these previous studies, ranging from two to six factors, fitted our sample and all the indicators of fit indicated a poor fit. Table 2 shows the result of the CFA analyses. In the discussion, we will elaborate on these findings according to the PSQ scale construction.

**Table 2.** CFA-Analysis of Factor Structures of PSQ-Op, 20 Items and PSQ-Org, 20 Items, Number of Factors Included.

	Number of factors		Chi-square	Degrees of freedom	CFI	RMSEA	SRMR
	PSQ-Op	PSQ-Org					
McCreary & Thompson, 2006 *n* = 560)	1	1	4349,447*	739	.727	.093**	.0734
SPO (*n* = 323)	1	1	2936,236*	739	.685	.096**	.0807
EWPE (*n* = 237)	1	1	2830,301*	739	.663	.110**	.0871
McCreary & Thompson, 2006 (*n* = 560)	1		1506,903*	170	.769	.119**	.0774
SPO (*n* = 323)	1		967,382*	170	.733	.121**	.0839
EWPE (*n* = 237)	1		983,585*	170	.690	.142**	.1013
Bélanger & Blanchette, 2022, (*n* = 560)	2		1117,761*	169	.836	.100**	.0678
SPO (*n* = 323)	2		787,983*	169	.793	.107**	.0775
EWPE (*n* = 237)	2		754,42*	169	.777	.121**	.0901
Kukić, Streetman, et al., 2021 (*n* = 560)	2		1296,004*	169	.805	.109**	.0726
SPO (*n* = 323)	2		844,528*	169	.774	.111**	.0786
EWPE (*n* = 237)	2		938,266*	169	.707	.139**	.0992
Kukić, Streetman et al., 2021 (*n* = 560)	2		1354,17*	169	.795	.112**	.0771
SPO (*n* = 323)	2		855,929*	169	.770	.112**	.0831
EWPE (*n* = 237)	2		973,383*	169	.693	.142**	.1001
Queirós et al., 2020 (*n* = 560)	2		1226,814*	169	.817	.106**	.0761
SPO (*n* = 323)	2		792,837*	169	.791	.107**	.0810
EWPE (*n* = 237)	2		933,74*	169	.709	.138**	.1075
McCreary & Thompson, 2006 (*n* = 560)		1	1583,118*	170	.767	.122**	.0992
SPO (*n* = 323)		1	1044,589*	170	.730	.126**	.0753
EWPE (*n* = 237)		1	796,454*	170	.768	.125**	.0754
Shane, 2010		6	903,148*	137	.863	.100**	.0630
SPO (*n* = 323)		6	598,124*	137	.847	0,102**	.0702
EWPE (*n* = 237)		6	526,101*	137	.841	.110**	.0672

*p < .001, **p < .05, RMSEA = Root Mean Square of Error Approximation, CFI= Comparative, Fit Index, SRMR = Standardised Root Mean Square Residual, SPO = Sworn police officer, EWPE = Employees without police education.

The second aim was to examine if a modification of the response scale by adding “Not Applicable” would reduce vulnerability and make the scales more robust. Table 3 (PSQ-Op) and IV (PSQ-Org) show the distribution of statistics regarding mean, standard deviation, number of answers (% of ‘Not Applicable’), and mean difference per item between SPO and EWPE in the PSQ-Op and PSQ-Org, respectively. All the ‘N/A’ scores are computed as ‘missing’ in Tables 3 and 4.

**Table 3.** PSQ-Op, Total and Stratified Distribution of Mean, Standard Deviation (SD), Probability of the Mean Difference Between SPO and EWPE (*p*).

	Total, *N* = 560			SPO, *N* = 323			EWPE *N* = 237			SPO/EWPE
	*Mean*	*SD*	*n (N/A %)*	*Mean*	*SD*	*n (N/A %)*	*Mean*	*SD*	*n (N/A %)*	*p*
Total	2.34	1.58		2.42	1.57		2.11	1.73		
1. Shift work	3.23	1.88	208 (62.9)	3.35	1.80	176 (45.5)	2.59	2.18	32 (86.0)	.037^a^
2. Working alone at night	2.38	1.91	117 (79.1)	2.41	1.92	92 (71,5)	2.28	1.86	25 (89.5)	N.S.
3. Overtime demands	2.27	1.63	302 (46.1)	2.30	1.62	218 (32.5)	2.19	1.68	84 (64.6)	N.S.
4. Risk of being injured on the job	2.13	1.41	271 (51.6)	2.20	1.38	211 (34.7)	1.52	2.13	60 (74.7)	N.S.
5. Work related activities on days off (e.g., court, community, events)	2.43	1.67	241 (57.0)	2.44	1.69	204 (36.8)	1.60	2.43	37 (84.4)	N.S.
6. Traumatic events (e.g., motor vehicle accident, domestic, death, injury)	2.32	1.51	249 (44.5)	2.37	1.51	214 (33.7)	2.00	1.51	35 (85.2)	N.S.
7. Managing your social life outside of work	2.56	1.63	489 (12.7)	2.68	1.61	306 (5.3)	2.36	2.56	183 (22.8)	.036^b^
8. Not enough time available to spend with friends and family	2.74	1.74	491 (12.3)	2.85	1.73	305 (5.6)	2.55	1.76	186 (21.5)	N.S.
9. Paperwork	2.71	1.61	436 (22.1)	2.80	1.61	303 (6.2)	2.51	1.58	133 (43.9)	N.S.
10. Eating healthy at work	2.18	1.54	489 (12.7)	2.35	1.58	303 (6.2)	1.90	1.42	186 (21.5)	.002^c^
11. Finding time to stay in good physical condition	2.88	1.68	530 (5.4)	3.04	1.67	316 (2.2)	2.64	1.68	214 (9.7)	.006^d^
12. Fatigue (e.g., shift work, overtime)	3.04	1.85	389 (30.5)	3.28	1.83	263 (18.6)	2.56	1.81	126 (46.8)	<.001^e^
13. Occupational related health issues (e.g., back pain)	2.49	1.70	461 (17.7)	2.51	1.66	294 (9.0)	2.45	1.76	167 (29.5)	N.S.
14. Lack of understanding from family and friends about your work	1.80	1.32	457 (18.4)	1.80	1.28	296 (8.3)	1.79	1.40	161 (32.7)	N.S.
15. Making friends outside the job	1.95	1.53	463 (17.3)	2.00	1.50	290 (10.2)	1.88	1.57	173 (27.0)	N.S.
16. Upholding a “higher image” in public	1.86	1.45	447 (20.2)	1.89	1.40	291 (10.0)	1.79	1.55	156 (34.2)	N.S.
17. Negative comments from the public	1.96	1.40	420 (25.0)	1.99	1.38	285 (11.8)	1.90	1.45	135 (43.0)	N.S.
18. Limitations to your social life (e.g., who your friends are, where you socialise)	1.82	1.28	460 (17.9)	1.89	1.28	297 (8.0)	1.68	1.27	163 (31.2)	N.S.
19. Feeling like you are always on the job	2.43	1.71	477 (14.8)	2.48	1.67	306 (5.3)	2.32	1.78	171 (27.8)	N.S.
20. Friends/family feel the effects of the stigma associated with your job	1.62	1.22	447 (20.2)	1.72	1.25	296 (8.3)	1.43	1.22	151 (36.3)	.020^f^

SPO = Sworn Police Officers, EWPE = Employees Without Police Education, SPO/EWPE = test of mean difference between SPO and EWPE, N/A = Not Applicable computed as missing, ^a^ = F(1, 206) = 4.430, ^b^ = F(1, 487) = 4.406, ^c^ = F(1, 487) = 10.041, ^d^ = F(1,528) = 7,520, ^e^ = F(1, 388) = 13.210, ^f^ = F(1, 445) = 5,456.

**Table 4.** PSQ-Org, Total and Stratified Distribution of Mean, Standard Deviation (SD), Probability of the Mean Difference Between SPO and EWPE (*p*).

	Total, N = 560			SPO, N = 323			EWPE, N = 237			SPO/EWPE
	Mean	SD	*n (N/A %)*	Mean	SD	*n (N/A %)*	Mean	SD	*n (N/A %)*	*p*
Total	2.47	1.7		2.54	1.65		2.34	1.76		
1. Dealing with the coworkers	2.02	1.47	500 (12.9)	1.92	1.26	309 (4.3)	2.17	1.73	191 (19.4)	N.S.
2. The feeling that different rules apply to different people (e.g., favouritism)	2,41	1.76	488 (4.8)	2.39	1.67	305 (5.6)	2.46	1.92	183 (22.8)	N.S.
3. Feeling like you always have to prove yourself to the organisation	2.59	1.75	505 (9.8)	2.56	1.71	311 (3.7)	2.64	1.82	194 (18.1)	N.S.
4. Excessive administrative duties	2.31	1.64	473 (15.5)	2.36	1.63	297 (8.0)	2.23	1.66	176 (25.7)	N.S.
5. Constant changes in policy/legislation	2.42	1.72	480 (14.3)	2.64	1.73	14 (4.3)	2.02	1.63	66 (27.8)	<.001^a^
6. Staff shortages	3.67	2.03	512 (8.6)	3.95	1.99	315 (2.5)	3.23	2.04	197 (16.9)	<.001^b^
7. Bureaucratic red tape	3.31	1.98	514 (8.2)	3.48	1.97	313 (3.1)	3.03	1.98	201 (15.2)	.011^c^
8. Too much computer work	2.51	1.71	515 (8.0)	2.71	1.70	313 (3.1)	2.19	1.68	202 (14.8)	<.001^d^
9. Lack of training on new equipment	2.35	1.70	495 (11.6)	2.40	1.68	312 (3.4)	2.25	1.72	183 (22.8)	N.S.
10. Perceived pressure to volunteer free time	2.01	1.56	448 (20.0)	2.11	1.56	293 (9.3)	1.84	1.55	155 (34.6)	N.S.
11. Dealing with supervisors	2.40	1.75	511 (8.8)	2.41	1.68	312 (3.4)	2.37	1.86	199 (16.0)	N.S.
12. Inconsistent leadership	2.59	1.92	497 (11.3)	2.57	1.89	308 (4.6)	2.61	1.99	189 (20.3)	N.S.
13. Lack of resources	3.75	2.05	527 (5.9)	4.10	1.99	316 (2.2)	3.23	2.03	211 (11.0)	<.001^e^
14. Unequal sharing of work responsibilities	2.89	1.89	522 (6.8)	2.93	1.82	313 (3.1)	2.82	1.99	209 (11.8)	N.S.
15. If you are sick or injured your coworkers seem to look down on you	1.68	1.40	449 (19.8)	1.61	1.21	294 (9.0)	1.82	1.69	155 (34.6)	N.S.
16. Leaders overemphasise the negatives (e.g., supervisor evaluations, public complaints)	2.22	1.76	471 (15.9)	2.34	1.80	305 (5.6)	2.01	1.66	166 (30.0)	.048^f^
17. Internal investigations	1.62	1.30	365 (34.8)	1.64	1.29	274 (15.2)	1.57	1.34	91 (61.6)	N.S.
18. Dealing with the court system	1.77	1.34	395 (29.5)	1.71	1.21	290 (10.2)	1.92	1.66	105 (55.7)	N.S.
19. The need to be accountable for doing your job	2.21	1.57	504 (10.0)	2.25	1.53	315 (2.5)	2.14	1.63	189 (20.2)	N.S.
20. Inadequate equipment	2.58	1.74	482 (13.9)	2.82	1.76	308 (4.6)	2.16	1.62	174 (26.6)	<.001^g^

SPO = Sworn Police Officers, EWPE = Employees Without Police Education, SPO/EWPE = test of mean difference between SPO and EWPE, N/A = Not Applicable computed as missing, ^a^ = F(1, 478) = 14.565, ^b^ = F(1, 510) = 15.491, ^c^ = F(1,512) = 6.457, ^d^ = F(1, 513) = 11.593, ^e^ = F(1,525) = 24.036, ^f^ = F(1,469) = 3.918, ^g^ = F(1,480) = 16.898.

The ‘N/A’ option was selected by both SPO and EWPE on all items in PSQ-Op. The SPO selected ‘N/A’ to a higher extent than expected given that the design of the PSQ was based on the content of the SPO working tasks. In our study, SPO rated operational stressors higher than EWPE. Only for ‘Fatigue’ were there significant (*p* < .001) mean differences between ratings from SPO and EWPE. The top three most stressful operational stressors were ‘Shift work’, ‘Fatigue’, and ‘Finding time to stay in good physical condition’. Interestingly ‘Shift work’ was also one of the two stressors where ‘N/A’ was most selected, the other being ‘Working alone at night’.

Regardless of educational background, the organisational stressors in PSQ-Org were found to be more stressful than the operational stressors in PSQ-Op. In five items (‘Constant changes in policy/legislation’, ‘Staff shortages’, ‘Too much computer work’, ‘Lack of resources’, and ‘Inadequate equipment’) we found significant (*p* < .001) mean differences between SPO and EWPE ratings.

The top three most stressful organisational stressors in our study were ‘Staff shortages’, ‘Lack of resources’, and ‘Bureaucratic red tape’. SPO rated the stressors in PSQ-Org higher than the EWPE. Overall, EWPE selected ‘N/A’ more often than the SPO in PSQ-Org. On the organisational scale ‘Internal investigations’ and ‘Dealing with the court system’ shared the highest use of ‘N/A’ by both groups.

## Discussion

Despite changes in the composition of police personnel (Lentz et al., 2020; Orosco & Gaub, 2022) most studies examining police working conditions are conducted on SPO, while EWPE are excluded. Intending to move research and practice on stress in the police forward and align results from research using the two scales of PSQ, the PSQ-Op and PSQ-Org, we undertook a psychometric evaluation of the PSQ combined with a deeper look at its construction and examined if a modification of the response scale would reduce vulnerability and make the measure more robust.

Our psychometric evaluation of the two scales of the PSQ, with the ‘N/A’-scores computed as ‘0’, resulted in poor fits first from the original factor structures of the PSQ (McCreary et al., 2017). This is consistent with the literature as in previous studies the tests of reported factor structures (Bélanger & Blanchette, 2022; Kukić, Streetman, et al., 2021; Kukić, Subošić, et al., 2021; McCreary & Thompson, 2006; Queirós et al., 2020; Shane, 2010) also resulted in poor fits. Previous studies have been inconclusive regarding a unified interpretation and comparison of results and outcomes on stress in the police between studies and countries. None of the studies including EWPE and applying PSQ (Jackman et al., 2021; Short, 2021; Varker et al., 2022) have conducted psychometric evaluations of the PSQ. We identified several variants of estimated factor exploration or confirmation endeavours in the literature with various numbers of selected items or different items in their models (Argo et al., 2021; Baek et al., 2021; Brunetto et al., 2022; Delgado Ramos & Vélez Vega, 2022; Fayyad et al., 2020; Louw & Viviers, 2010; Queriós et al., 2020; Rasdi et al., 2014; Sagar et al., 2014, 2015; Shane, 2010). As studies using the PSQ have proliferated across police organisations and countries the lack of shared understanding of factor structure may result from cultural differences (Orosco & Gaub, 2022), or differences in the organisations of policing or policing tasks (Bélanger & Blanchette, 2022).

Based on our findings, we suggest this may also be due to the nomological status of the PSQ, i.e., whether organisational and operational stress should be understood as formative or reflective concepts. In light of the theory on models of scale construction (Diamantopoulos & Siguaw, 2006), the measured stressors in PSQ-Op and PSQ-Org are either developed to investigate stress as an underlying latent factor or as an aggregation of demanding operational or organisational police work tasks. The former requires a reflective and the latter a formative approach. Reflective scale construction is based on intercorrelations between the items and internal data consistency and predicted variables, while a formative index emphasises the role of the items as predictors (Diamantopoulos & Siguaw, 2006).

The results of our study, where EWPE are included and ‘N/A’ added to the response scale, replicate results of previous studies with or without EWPE included (Delgado Ramos & Vélez Vega, 2022; Jackman et al., 2021; Kukić, Subošić, et al., 2021; Shane, 2010; Short, 2021; Varker et al., 2022); organisational stressors are rated higher than operational stressors, and the most stressful operational and organisational stressors are the same. These findings confirm that neither the option of ‘N/A’ added to the response scale in our study nor the inclusion of EWPE interferes with established results. As expected, and aware of the known shortcomings of rating references in all self-report questionnaires, we found few significant mean differences in item responses between SPO and EWPE. The differences found may be an expression of the content of the PSQ items in terms of relevance, representativeness, and technical quality (Boateng et al., 2018) being influenced by time and context affecting stressor expositions (Bélanger & Blanchette, 2022; Orosco & Gaub, 2022).

The option of ‘N/A’ was however selected more frequently than expected, even by SPO in PSQ-Op, suggesting general statements about the main sources of stress in the police, should be treated with caution. Adding ‘N/A’ to the questionnaire will increase the number of respondents and thereby increase the statistical power of the assessment. As in other studies with EWPE included (Jackman et al., 2021; Varker et al., 2022), the SPO also rated stressors in both PSQ scales higher than EWPE. One potential explanation may be that PSQ-Op items are influenced by time and the national context of the application. Thus, despite lack of shared understanding of factor structure, our findings do resonate with the consistent finding on stress in the police that organisational stressors are perceived as more troublesome than operational. This also holds when adding the option of ‘N/A’ and including EWPE.

Based on our findings we encourage researchers to include EWPE in studies of police stress when applying the PSQ. We also recommend including information from this group of police employees in practical preventative and intervention work targeted at improving health and wellbeing in the police.

## Conclusions

This study undertook a psychometric evaluation of the PSQ (McCreary & Thompson, 2006) and investigated if a modification of the response scale would make the measure more robust. Lack of shared understanding of factor structure hampers the possibility of interpreting and comparing results and outcomes on stress in the police between studies and countries, advancing research on stress in the police, and developing efficient implications of practice. Based on our results, we suggest framing the PSQ scales as formative. This implies viewing the indicators (items) as defining characteristics of the construct, rather than a reflective framing where the construct, i.e., operational and organisational stress respectively, define characteristics of the indicators. Operational and organisational stress are thus not latent variabels and should in future research not be treated as such. The results, in light of scale construction, showed that the two scales of the PSQ have some fit with the original factor structures of the PSQ for both SPO and EWPE. There are however several difficulties. The modification (i.e., ‘Not Applicable’) offers extended information and thereby provides an influential elaboration of the PSQ in future studies of stress in the police and the practical implication, e.g., targeted interventions, to be drawn from these results.

## Limitations

For the credibility of our results, this study is based on a random sample of police employees from official statistics in the NPD. Still, the response rate (RR) is low. Regardless of RR, transparency is recommended to ensure the assessment of validity (Holtom et al., 2022). In our case, the study sample holds good representativity and statistical power. The low RR may be due to difficulties with the digital survey log-in solution, and the length. As this study applied random sampling and has performed data cleaning, data quality, and participant identity checks, the study has a ‘functional RR’ (Holtom et al., 2022). That is, its results provide reasonable inferences for stress among police employees, regardless of educational background.
